# d-matrix – database exploration, visualization and analysis

**DOI:** 10.1186/1471-2105-5-168

**Published:** 2004-10-28

**Authors:** Dominik Seelow, Raffaello Galli, Siegrun Mebus, Hans-Peter Sperling, Hans Lehrach, Silke Sperling

**Affiliations:** 1 Vertebrate Genomics, Max-Planck-Institute for Molecular Genetics, Ihnestr. 73, 14195 Berlin, Germany; 2 Pediatric Cardiology, German Heart Center, Augustenburger Platz 1, 13353 Berlin, Germany

## Abstract

**Background:**

Motivated by a biomedical database set up by our group, we aimed to develop a generic database front-end with embedded knowledge discovery and analysis features. A major focus was the human-oriented representation of the data and the enabling of a closed circle of data query, exploration, visualization and analysis.

**Results:**

We introduce a non-task-specific database front-end with a new visualization strategy and built-in analysis features, so called d-matrix. d-matrix is web-based and compatible with a broad range of database management systems. The graphical outcome consists of boxes whose colors show the quality of the underlying information and, as the name suggests, they are arranged in matrices. The granularity of the data display allows consequent drill-down. Furthermore, d-matrix offers context-sensitive categorization, hierarchical sorting and statistical analysis.

**Conclusions:**

d-matrix enables data mining, with a high level of interactivity between humans and computer as a primary factor. We believe that the presented strategy can be very effective in general and especially useful for the integration of distinct data types such as phenotypical and molecular data.

## Background

d-matrix, originally designed with cardiovascular clinical and molecular genetic data in mind, is a generic database front-end that can be used to explore, visualize and analyze different typologies of datasets.

Both the generation and the analysis of genome, transcriptome and proteome data are becoming increasingly widespread, and these data must be merged to generate a molecular phenotype. Moreover, the correlation between molecular and phenotypical data requires acquiring both with comparable profoundness leading to the development of large and small scale databases holding both information [1-3]. In the same line, we developed a CardioVascular Genetic database (CVGdb), storing the detailed clinical phenotype of patients with congenital heart diseases as well as molecular data such as gene expression analysis results [4] and genotypes. However, querying and analyzing the stored data to uncover the valuable information hidden in the databases are difficult tasks. With some exceptions, these are approached by a two-step procedure, in which a database specific front-end serves the query and extraction of data, which are subsequently imported in stand-alone analysis tools for visualization, mining and statistics [5-11]. Moreover, the visualization and mining tools frequently focus on presenting overall views of data sets for a specific task and seldom permit single-case addressability or have drill-down capability. In today's systems, the perceptual abilities of human users are only used to a limited extend. We believe that it is essential to make users part of the overall process through computer support of their intelligence, creativity and perceptual abilities. Hence, a major research challenge is to find human-oriented forms of representing information and enabling rapid interaction between humans and computers in the query, visualization and analysis process [12].

It is not the purpose of this paper to survey the various solutions available to query, visualize and mine data, but rather to illustrate how such concepts could be combined usefully within one software tool. Here, the layout should not only preserve the structure of the information, it should also convey the quality of the distribution of the values contained in the database. The features of the display should then be designed to highlight those regularities, patterns or dependencies that are not easily detectable with an ordinary front-end.

One visual representation, which motivated the graphical display of the tool we describe here, is the data matrices handled in microarray studies, in which rows in the matrices typically represent genes and columns individual samples [13]. Rather than showing a numerical 'spreadsheet', it is convenient to display microarray data in such matrices, which indicate varying expression levels in a grid of varying colors.

With d-matrix we propose a generic front-end solution capable of extracting, exploring, visualizing and analyzing complex data. The software can be interfaced with the most common relational database management systems without any intervention on the schema or pre-processing phase. As the name suggests, the visual model proposed has the form of a matrix. Its elements are boxes whose colors show the quality of the underlying information. The granularity of the data display allows consequent drill-down, i.e. the user is able to focus the observation on a single data point. In addition, value frequency bars are available to present compact overviews. It also offers the possibility to define categories using context-sensitive rules and to assign colors to classes. The direct implementation of a broad range of descriptive and advanced statistics together with a hierarchical sorting feature permits user-defined exploration of the data.

## Implementation

### Data Model

The process of developing a uniform web interface for disparate data sources is a complex task because of the variability in the data models that underlie each source. To enable an effective two-dimensional display, the d-matrix model consists of a three-level tree. For the representation of a large database schema requiring a higher number of levels, several d-matrix instances can be built on the same database.

Within the proposed model, the main table addressing the objects of a study is considered as the root, the first level of the tree. The second level consists of tables that are joined with the root by means of its primary key and the third level consists of tables that are further joined with the ones at level two. In particular, the dependency of the root table with the second level tables can be either one-to-many or one-to-one, while the dependency of the second level table with the third level ones can be either many-to-one or one-to-one. To apply different query and visualization rules each branch of this tree is defined as a data group characterized by the same storing strategy. In cases where Entity-Attribute-Value (EAV) tables are interfaced, the Entity must correspond to the main ID.

**Figure 1:**
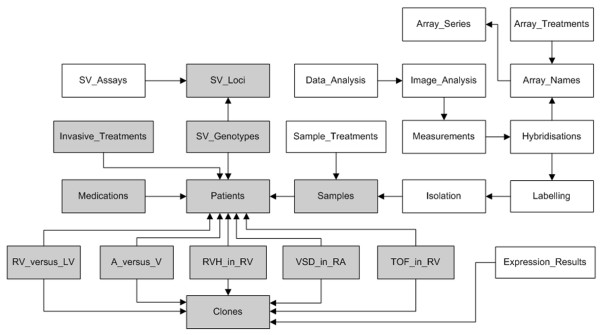
**Relational database schema of the CardioVascular Genetics database (CVGdb) **Tables are represented as boxes and foreign keys constraints as arrows. Grey boxes mark the schema subset interfaced in d-matrix. SV – Sequence Variations; RV_versus_LV, A_versus_V, RVH_in_RV, VSD_in_RA and TOF_in_RV are tables containing gene expression results [4].

**Figure 2:**
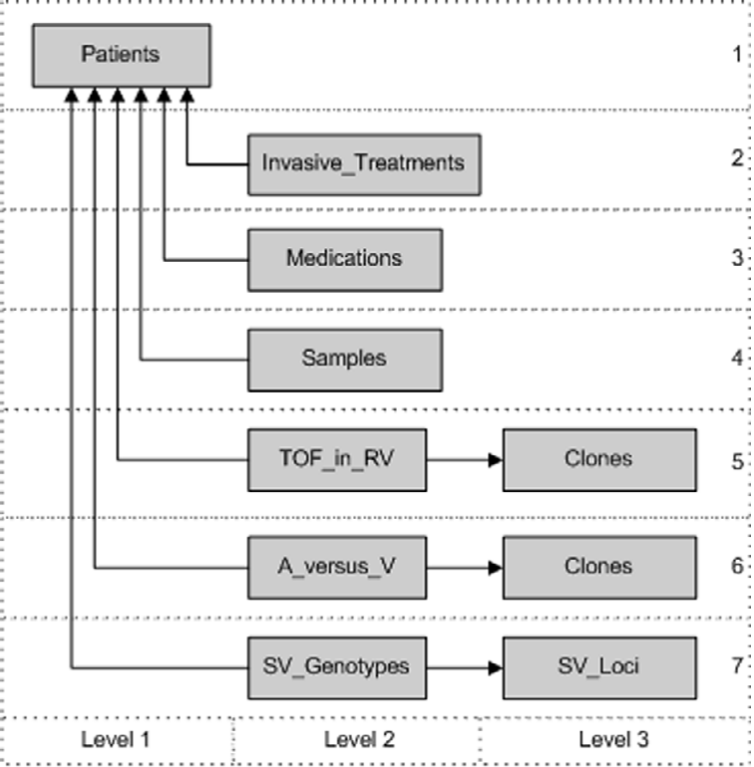
**Data Model **Shown is an excerpt of the three-level tree for interfacing d-matrix with CVGdb. The table "Patients" defines the root of the tree. Each branch refers to a defined data group consisting of one or two tables, respectively.

As an example, we can refer to the CardioVascular Genetics database (CVGdb) schema set up by our group (Figure 1). Here, we selected the table Patients as the root of the tree, so that the CVGdb instance main ID is the Patients primary key. This selection is arbitrary and one could also choose Clones or Hybridizations, thereby focusing on different aspects of the overall dataset. In Figure 2, data groups and tree levels are represented. The data groups 2 to 4 address the EAV tables Invasive_Treatments, Medications and Samples; the groups 5 to 6 contain the same table Clones joined with different tables containing gene expression analysis results [4]; whereas the last group is built by two tables describing sequence variations (SV).

### Data selection and query

**Figure 3:**
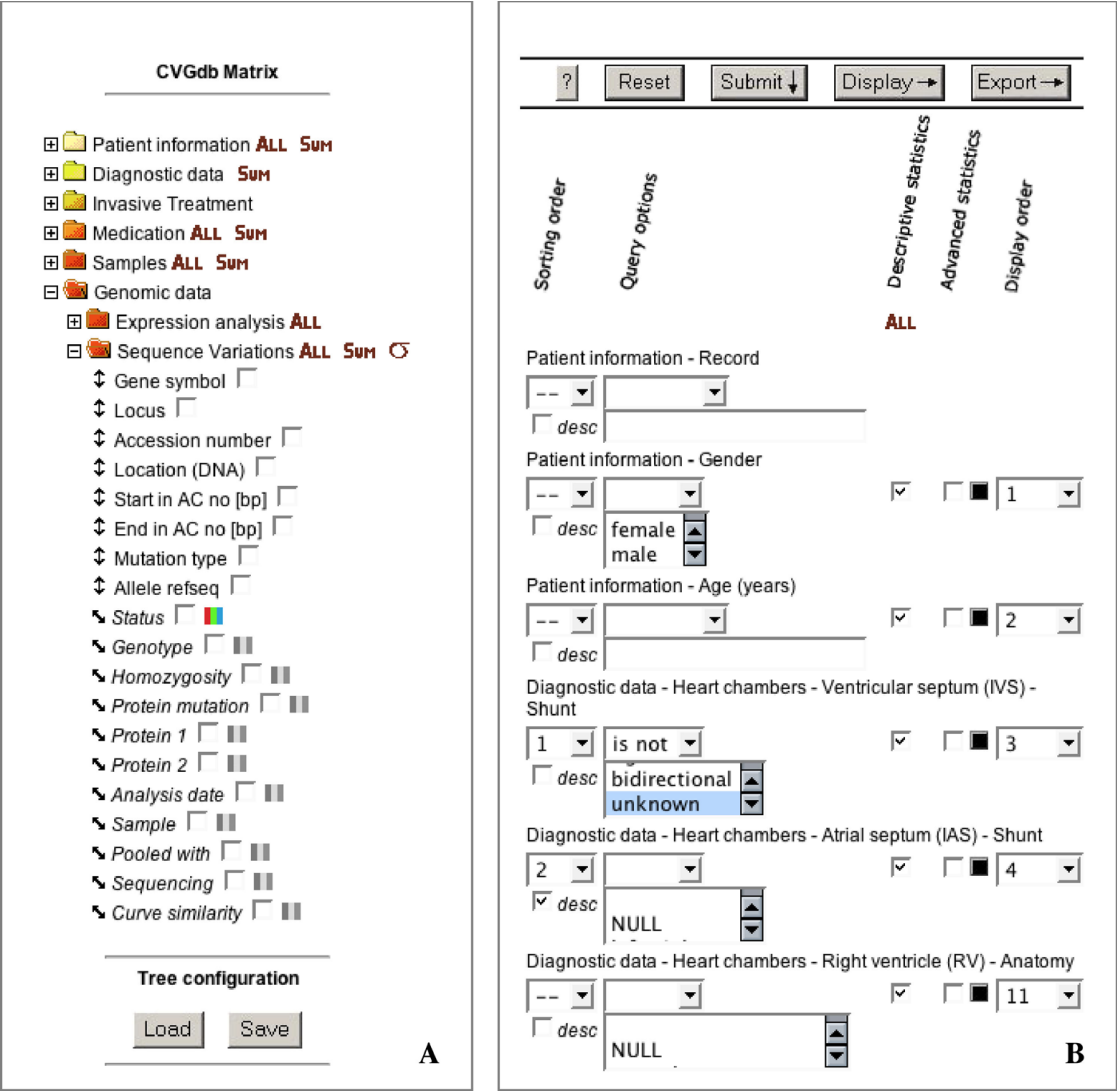
**Data selection and query **Within the data selection schema (A) users can choose all nodes they want to be included in the query. If a data group consists of two tables, the nodes are represented by vertical arrows for the first table and diagonal arrows for the second. The attribute on which the query display is focused can be selected by the three-banded icons, which switch from black-white to color and vice versa upon selection. Furthermore, trees can be saved and reloaded for subsequent analysis. Upon selection all nodes are listed in a secondary form (B), where query conditions, display and sorting order as well as the implementation of descriptive and advanced statistic can be specified. In addition to the graphical output, the query can be exported as a text of XML file.

The schema is presented to the user in a structure recalling a file system selector (Figure 3A). Nodes represent attributes or value attributes that can be optionally divided further into more folders without any depth limitation. Collecting nodes in visually distinct entities becomes a necessity when coping with a large number of attributes. To obtain a quantitative measure of the information that is contained within groups of nodes, a *summary node *can be included in the query form. For each value of the x-axis, the values of the summary nodes are computed by counting the nonempty nodes in the respective folders.

The query process consists of two steps. First, the users select all nodes they want to be included in the query (Figure 3A); second, these are listed in a query form where conditions and analysis features can be specified (Figure 3B). To visually distinguish between nodes referring to data belonging to single-table data group and two-table data groups, single-table group nodes are represented as sheet-like-icons, whereas two-tables data group nodes are represented by double-arrow-like icons: diagonally oriented for the nodes that belong to the second level tables, and vertically oriented for the nodes that belong to the third level tables (Figure 3A). The attribute on which the query display shall be focused can be selected by means of the three-banded icons placed on the right side of the nodes. For each of the nodes, the query form permits the definition of sorting order and direction (ascendant/descendent), values and operators for query conditions, display order and parameters for statistical evaluations. The value cell is not shown if the node itself is an attribute value. Alternatively to the matrix view of the query result, the user can optionally export the resulting dataset in form of text or XML (Figure 3B).

### Data visualization

**Figure 4:**
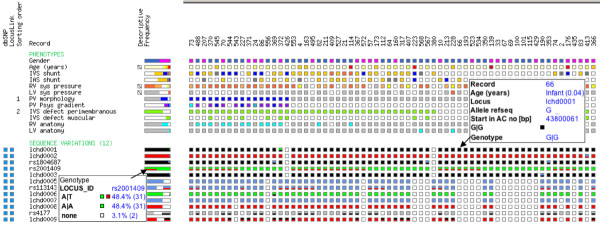
**Graphical output of d-matrix **The graphical output consists of the matrices itself, the description of the nodes displayed, a prospect of statistical evaluations and hyperlinks to external resources. Each matrix corresponds to a single data group (Phenotypes; Sequence variations). The x-axis of the matrix is defined by the main ID (Record) and the y-axis by the nodes displayed. The terms like "Gender", "Age (Years)" and "IVS Shunt" are descriptive names for the respective column names GENDER, AGE_YEARS and IVS_SHUNT of table PATIENTS; terms like "Ichd0001" and "Ichd0002" refer to locus names, values of the column LOCUS_ID of table SEQ_VAR_LOCI. The matrix is built by colored boxes coding for the meaning of the information itself, which is further described in the pop-up window (as shown for Record 366 and Ichd0009). Frequency bars and boxes for descriptive statistics are displayed. Numbers are reflecting the sorting order, whereas blue boxes at the left border hold the hyperlinks.

The graphical output of d-matrix consists of two-dimensional matrices, whose colored boxes code the meaning of the underlying information, the description of the chosen nodes and a prospect of statistical evaluations (Figure 4). The display of the data is determined by the data dimensionality. The main ID corresponds always to the x-axis of the matrix. To permit the display of single and multiple dependencies with the main ID, the y-axis shows either node descriptions or node values.

In cases of single dependency each data point is represented by one box of the matrix. If there is a multiple dependency (two-table data groups), subsequently more rows for each value of a single node are displayed. EAV data groups can lead to both single or multiple dependency; in the second case the entries are aggregated in one matrix box. In Figure 4 the tuples of the data group "PHENOTYPES" addressing the table Patients are displayed in the first matrix. Each tuple corresponds to a column whereas row headers are node descriptions. The tuples of the data group "SEQUENCE VARIATIONS" addressing the tables SV_Genotypes and SV_Loci are aggregated column-wise and grouped by the main ID. Here, there is more than one tuple for each column whereas row headers are values of the node Locus ID. Hence, each column of boxes on the matrix display represents an aggregation of more than one tuple of the query result. Following data mining terminology, we can say that in d-matrix *cases *(and aggregations of them) are represented column-wise.

When the matrix oversize the available space, the use of two distinct scrollbars lets the user move the data matrix horizontally and vertically. The general overview is given together with the advantage of single-case addressability, i.e. each case (tuple) representation is entirely visible and its components clearly distinguishable.

The display is obtained as a group of images (generated using the Perl GD module and stored as temporary files), each in a separate HTML DIV container, which can be moved independently.

### Drill-down

The matrix display represents a summarized view of the query. Each box holds three levels of detail: first, the coordinates that uniquely identify the box position and represent two units of information; second, the color that corresponds to either a single value or a category; third, the hidden content of the box obtained by drill-down, which gives all remaining information for that box.

In the d-matrix display the drill-down can be obtained for each box in form of a pop-up window (Figure 4). The content structure of this new window varies according to the data group to which the box belongs, although it always contains the value that is substituted by its color code together with the underlying node description. Further supplementary data can be included from attributes of the same data group.

It is possible to add further detail by the mean of hyperlinks to grant access to remote databases, external analysis results and multimedia documents (Figure 4), or even to trigger further analysis processes.

### Schema interface and configuration

The software requires four configuration files: the data definitions file that is needed to connect d-matrix with the relational schema, a database settings file storing the information to access the database, a color file for the definition of the colors used in the matrix and a general server settings file. Every configuration file is maintained as plain text to permit easy access and modification.

The structure of the data definitions file must reflect the hierarchy in which the metadata (relational schema definition) have to be organized on the screen, while its textual content depicts a level of abstraction (*definitional abstraction*) [14] between the database physical representation and the human-comprehensible view of the data. Therefore, the data definitions file reflects the subdivision of the database schema in data groups. For each group the table attributes, information about identifiers, joining conditions as well as aggregation (where needed), display settings and the content of the pop-up window have to be defined. User-defined human-intelligible terms can be assigned for any term used in the database. Besides the attributes' names, types and descriptions, it is possible to define categories, orderings and associations with colors. It is important to notice that the rules that define categories can even involve other attributes of the same data group. This context-sensitive categorization, intended as a *qualitative abstraction *[14], allows the concurrent representation of two layers of information.

For each attribute value, value range or defined category, rules can be given to assign its respective color. This leads to a common method to visualize both discrete and continuous variables. In addition, categorized numeric values can be treated as categorical in specific contexts like sorting and statistics. Furthermore, colored boxes can be composed by combining the values of two nodes, which enables, for example, the visualization of both Alleles within horizontally split boxes for sequence variations (Figure 4).

Several data definitions files (each defining a separate d-matrix instance) can independently coexist on the same server for the same or different database systems and schemata.

### Visual data mining and statistical analysis

d-matrix permits consecutive data-filtering operations that – as a whole – can be seen as a single user-driven data mining session. A compact and information-dense graphical outcome, context-sensitive categorization, hierarchical sorting and drill-down enable this mining process. Frequency bars give an overview of the overall queried dataset whereas box plots improve the visual perception of the data distribution. A key feature within the mining process is the opportunity to obtain different views of a single data set rapidly in parallel using different browser windows. Here, the interactivity becomes a primary factor and is supported by the human-oriented representation.

A wide range of descriptive statistics and statistical tests is directly accessible. This permits statistical evaluation of the correlation between attributes and determination whether it is reasonable or not to assume that a sample fits to a specific distribution. For numerical values it is possible to perform up to ten different statistical tests, while for non-numerical entities (Boolean and categorical data) the Chi-square and Fisher exact tests are available. The user interface automatically performs a selection of attributes and tests according to their respective compatibility.

In addition to directly implemented tests, external data analysis environments like R [15] or user defined routines can be easily interfaced. The results of the tests, together with the descriptive statistics, are displayed at the side of the matrix and colors of the boxes reflect the results (e.g. significance) of the tests.

### CardioVascular Genetics database (CVGdb)

**Figure 5:**
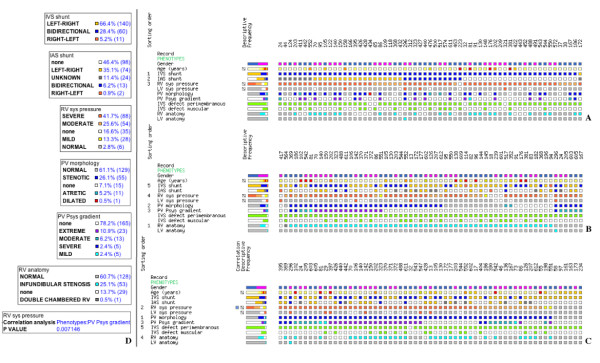
**Example of a data exploration session for CVGdb **Shown are the first 61 of 211 records that meet the query condition "IVS shunt" is not "Null" focusing on different views of the data given by different sorting options (A, B, C). To provide information about the color code as well as the overall query output, pop-up windows for frequency bars of sorted nodes are shown (D). Further, the pop-up window for the correlation analysis between 'RV sys pressure' and PV Psys gradient' is displayed (D). See text for detailed description of the observed cluster.

For interfacing d-matrix with the CVGdb, we assigned categories if appropriate and colors to more than 700 nodes. Figure 5 shows an example of a single user-driven data mining session, which was initiated with the aim to discover cardiac phenotype features associated with shunts abroad the interventricular septum (IVS shunt). Therefore, the only query condition specified is that "IVS shunt" is not "NULL". This condition is fulfilled by 211 out of 560 IDs stored to date. In addition, a subset of nodes referring to phenotype descriptions physically surrounding the interventricular septum has been chosen to be displayed. To structure the display, hierarchical sorting has been applied to the 'IVS shunt' and an arbitrary selection of other nodes. Viewing the entrance matrix (Figure 5A), one could easily recognize data clusters such as the relation of the category 'bidirectional' of the 'IVS shunt' (blue boxes) to categories of interatrial septum shunts (IAS shunt) and right ventricular systolic pressure 'RV sys pressure'. Almost all patients with a bidirectional 'IAS shunt' are also characterized by a bidirectional 'IVS shunt'. Furthermore, the majority of bidirectional 'IVS shunt' is associated with severe 'RV sys pressure', whereas the non-sorted nodes pulmonary valve morphology (PV morphology), pulmonary valve systolic pressure gradient (PV Psys gradient) and right ventricular anatomy (RV anatomy) are distributed in a questionable co-occurrence to each other in this first matrix. For further evaluation, we focus on the 'RV anatomy' or the 'PV morphology' chosen as the first sorted nodes in the second and third matrix (Figure 5B,5C), respectively. By using the tree save/reload option to retrieve these new matrices, only the sorting criteria needed to be modified to obtain different views on the same data set in parallel using three browser windows. Hence, the frequency bars remain the same in all visualization sessions. Now it becomes clear that more than half of the patients with infundibular stenosis (RV anatomy) show a stenotic 'PV morphology', which by itself is highly associated with an extreme 'PV Psys gradient'. Applying the correlation analysis implemented in d-matrix, the significance of the correlation of the 'PV Psys gradient' with the 'RV sys pressure' could be verified (Figure 5D). The described data are available for the exploration using d-matrix at the web supplement.

Finally, the session explained is just one out of several examples in which d-matrix proved to be highly effective for the visualization of regularities and dependencies within the CVGdb data. Moreover, based on the general visualization concept, d-matrix provides an integration between clinical and genetic information that is crucial for the correlation of phenotypical and molecular data (Figure 4).

### Other applications

With respect to an ongoing project on gene regulation, we found it very convenient to visualize potential transcription factor binding sites (TFBS) in promotor sequences by interfacing d-matrix [16]. Here, the nucleotides are used as the main ID (x-axis) and the TFBS are consequently displayed at the y-axis. This allows a much higher level of interactivity than a usual figure output. One could easily have different views of the data set by sorting or parallel display of different information, like color coded core or matrix match similarities.

To demonstrate the versatility of the software, we further interfaced d-matrix with a database that represents the periodic table of the elements [16]. Although we did not expect unusual or unexpected regularities in such a simple case, it was easy to obtain a matrix that shows the well-known dependency between Atomic Number, Atomic Mass and Energy Levels and the obvious lack of available information about elements with seven energy levels, which are the most unstable and rare.

The interfacing with both dataset required only one working day for each.

## Results and discussion

We have presented d-matrix, a non-task-specific database front-end with a new visualization strategy with embedded analysis features.

The graphical outcome of d-matrix consists of colored boxes arranged in matrices; it permits single-case addressability with further drill-down capability. Together with the hierarchical sorting and statistical feedbacks, d-matrix enables consecutive data-filtering operations that – as a whole – can be considered as a single data mining session. Also, the result of such a session can be exported for further study. For a qualitative evaluation of d-matrix, one should not only focus the attention on the final display, which only represents the end product of a sequence of user-driven data exploration sessions. The high level of interactivity that our approach offers is indeed a primary factor; with d-matrix, the communication between human and computer is a rapid interaction.

The future development of d-matrix will focus on the implementation of clustering algorithms to be executed before display. Furthermore, we envisage the design of instruments to inquire metadata to maximize the quantity of information that will be eventually displayed and analyzed [17]. In addition, a user-friendly way to interact with configuration files will be granted by specific CGI scripts leading to a further reduction of the time to interface d-matrix with relational schemata.

An inquiry of the solutions reported to date for data exploration, visualization and analysis resulted in an approximate distinction between reports about efforts for database development with their task specific front-end solutions and stand-alone data analysis, visualization and mining tools. In our view, d-matrix stands in between those two groups and aims to combine features of both efforts, which we believe can be very effective and useful in general and especially for the association of distinct data types such as phenotypical and molecular data. As a front-end, it does not require complex installation processes or maintenance, and it is suitable for multi-user remote access. As a visual data mining tool, it gives an effective display that allows the detection of exceptions, trends, regularities, clusters and dependencies, as well as incomplete or erroneous data.

## Availability and requirements

**Project name: **d-matrix

**Project home page: **

**Operating system(s): **Platform independent

**Programming language: **Perl

**Other requirements: **d-matrix was successfully interfaced to Oracle 8i, MySQL, Microsoft Access and text-based databases and is compatible with recent JavaScript-enabled browsers.

**License: **d-matrix is available on request from the author. To academic institutions d-matrix is available for a fee of 250 Euro that is intended to cover our costs of distribution and maintenance.

## Authors' contributions

DS developed the first generation of d-matrix and carried out the main programming work. RG is the current maintainer and carried out the main implementation. SM and HPS participated in the design, testing and quality control. HH participated in the conceptual design. SS conceived the development of d-matrix, managed and participated in its design and implementation.

## References

[B1] Fredman D, Munns G, Rios D, Sjoholm F, Siegfried M, Lenhard B, Lehvaslaiho H, Brookes AJ (2004). HGVbase: a curated resource describing human DNA variation and phenotype relationships. Nucleic Acids Res.

[B2] Genome Web. http://www.hgmp.mrc.ac.uk/GenomeWeb/.

[B3] Nadkarni PM (2003). The challenges of recording phenotype in a generalizable and computable form. Pharmacogenomics J.

[B4] Kaynak B, von Heydebreck A, Mebus S, Seelow D, Hennig S, Vogel J, Sperling HP, Pregla R, Alexi-Meskishvili V, Hetzer R, Lange PE, Vingron M, Lehrach H, Sperling S (2003). Genome-wide array analysis of normal and malformed human hearts. Circulation.

[B5] Walker AJ, Cross SS, Harrison RF (1999). Visualisation of biomedical datasets by use of growing cell structure networks: a novel diagnostic classification technique. Lancet.

[B6] Falkman G (2001). Information visualisation in clinical Odontology: multidimensional analysis and interactive data exploration. Artif Intell Med.

[B7] Shao Q, Li Y, Campbell E, De Boer ES, Laginestra E, Statzenko A (2003). Statistical visualization for data exploration: a case study on Sydney Olympic Park. Chemosphere.

[B8] Gilbert DR, Schroeder M, van Helden J (2000). Interactive visualization and exploration of relationships between biological objects. Trends Biotechnol.

[B9] Grinstein G, Trutschl M, Cvek U (2001). High-Dimensional Visualizations. 7th Data Mining Conference-KDD 2001: San Francisco, California.

[B10] Wegman EJ (2003). Visual data mining. Stat Med.

[B11] Rost U, Bornberg-Bauer E (2002). TreeWiz: interactive exploration of huge trees. Bioinformatics.

[B12] Keim D, Kriegel HP (1994). VisDB: Database Exploration using Multidimensional Visualization. IEEE Computer Graphics and Applications: 1994.

[B13] Eisen MB, Spellman PT, Brown PO, Botstein D (1998). Cluster analysis and display of genome-wide expression patterns. Proc Natl Acad Sci U S A.

[B14] Lavrac N, Keravnou E, Zupan B (2000). Intelligent Data Analysis in Medicine.

[B15] Ihaka R, Gentleman R (1996). Language for Data Analysis and Graphics. J of Comp and Graphical Stats.

[B16] d-matrix web supplement. http://www.molgen.mpg.de/~heart/index_dmatrix.html.

[B17] Weiner M, Sherr M, Cohen A (2002). Metadata tables to enable dynamic data modeling and web interface design: the SEER example. Int J Med Inform.

